# Arterial Enhancement Fraction-Spectral CT-Based Model as Part of Prediction Model in BRAF^V600E^-Positive Papillary Thyroid Carcinoma

**DOI:** 10.3390/diagnostics15212817

**Published:** 2025-11-06

**Authors:** Bi Zhou, Liang Lv, Ya Zou, Zuhua Song, Jiayi Yu, Xiaodi Zhang, Dan Zhang

**Affiliations:** 1 Department of Radiology, Chongqing General Hospital, Chongqing University, Chongqing 401147, China; zhoubifangshe@foxmail.com (B.Z.); lvliang506@sohu.com (L.L.); 130546@cqu.edu.cn (Y.Z.); szuh2013@foxmail.com (Z.S.); yujiayi511@sohu.com (J.Y.); 2 Department of Clinical and Technical Support, Philips Healthcare, Chengdu 610041, China; megy.zhang@philips.com; 3 Department of Radiology, the Second Affiliated Hospital of Chongqing Medical University, Chongqing 400010, China

**Keywords:** arterial enhancement fraction, dual-layer detector spectral CT, papillary thyroid carcinoma, BRAF mutation, nomogram

## Abstract

**Objectives**: The BRAF^V600E^ is the most common oncogene in thyroid cancer and is associated with the aggressiveness of papillary thyroid carcinoma (PTC). The aim of this study was to investigate the effectiveness of the arterial enhancement fraction (AEF) and dual-layer detector spectral computed tomography (DLCT) parameters for predicting the BRAF^V600E^ mutation in PTC. **Methods**: A total of 237 patients with PTC who underwent DLCT and BRAF^V600E^ mutation detection (mutant group: *n* = 187; wild group: *n* = 50) were retrospectively reviewed. The receiver operating characteristic curves evaluated the effectiveness of the prediction models based on the significantly different variables using logistic regression analysis. The nomogram of the prediction model with the highest AUC in the validation cohort was constructed. **Results**: The AUCs of the DLCT+ Hashimoto’s thyroiditis (HT) and AEF + DLCT + HT prediction models were 0.901 and 0.896, respectively, in the training cohort and 0.801 and 0.853 in the validation cohort. The calibration curve revealed the good agreement between the prediction results and the actual observations using the AEF + DLCT + HT model. The DCA demonstrated that the model can provide net benefit for all threshold probabilities. **Conclusions**: As an effective and visually noninvasive prediction tool, the AEF + DLCT + HT-based nomogram presented satisfactory effectiveness in preoperatively predicting the BRAF^V600E^ mutation in PTC.

## 1. Introduction

Thyroid cancer is the most prevalent endocrine neoplasm worldwide. Papillary thyroid carcinoma (PTC) is the most common subtype, accounting for approximately 80% of all thyroid cancers [1]. However, PTC with aggressive features, such as aggressive variant, regional metastases, extrathyroidal extension, and distant metastases, suggests a poor prognosis [2,3,4]. The B-type Raf kinase (BRAF) is an oncogene in human cancer [5]. The BRAF^V600E^ mutation leads to malignant transformation and the potential loss of differentiation capacity. Previous studies revealed that the BRAF^V600E^ mutation is a prognostic marker that is associated with high TNM stage, extracapsular invasion, and the cervical lymph node (LN) metastasis of PTC [6]. In addition, patient-derived PTC organoids with the BRAF^V600E^ mutation showed a drug response to BRAF inhibitor-based combinations [7]. The 2015 American Thyroid Association Guideline also included the BRAF^V600E^ mutation as a risk stratification factor [8]. Therefore, the preoperative detection of the BRAF^V600E^ mutation is important for appropriate surgical planning and comprehensive therapy selection in patients with PTC.

Currently, ultrasound (US) is the first-choice tool for the assessment of thyroid nodules. However, US depends on operators’ experience and is limited in the face of the widely distributed cervical LN disease, insufficiently visualizing possible neck nodules (high thyroglobulin but negative result with US) and mediastinal metastases. In contrast, CT presented advantages in detecting metastases in the central cervical region, mediastinum, and behind the trachea. Moreover, CT could provide a definitive surgical plan with the adequate preoperative mapping [8,9]. Therefore, the CT scan is increasingly performed for the preoperative assessment of the thyroid nodules.

This study focuses on two advanced CT-derived techniques, arterial enhancement fraction (AEF) and dual-layer detector spectral CT (DLCT), to address the need for the non-invasive prediction of the BRAF^V600E^ mutation. The AEF is defined as the ratio of absolute enhancement in the arterial phase to that of the venous phase in CT examinations. It correlates with tumor perfusion and has exhibited promising value in diagnosing cervical LN metastasis in PTC and assessing the treatment outcome of liver diseases [10,11]. Previous studies have reported higher microvascular density in BRAF^V600E^ mutant colorectal cancer tissues and higher protein levels of angiogenic factors in undifferentiated thyroid carcinoma (ATC) [12,13]. Therefore, we hypothesize that the hemodynamic feature in BRAF^V600E^-mutant PTC may differ from that of wild-type BRAF PTC, and the AEF may distinguish the difference. Dual-energy computed tomography (DECT) is a novel CT technique that can produce multiple quantitative parameters, such as virtual monoenergetic images (VMI), iodine concentration (IC), effective atomic number (Zeff), and so on. The energy parameters derived from DECT can improve tumor conspicuity [14,15], differentiate malignant tissue from normal or inflamed tissue, and preoperatively predict the early recurrence of carcinoma [16,17]. The single X-ray beam was decomposed into high- and low-energy components simultaneously via the two predefined detector layers of DLCT, which is the basis of the dual-energy technique in DLCT. Previous studies have demonstrated that the quantitative parameters of DLCT, or their combinations, have differential diagnosis values for thyroid nodules and cervical LN metastasis, and even for micro-nodules [18,19].

To our knowledge, there have been no published studies predicting the BRAF^V600E^ mutation in PTC using the AEF combined with dual-energy CT. Consequently, this study evaluated the potential benefit of the AEF and DLCT in predicting the BRAF^V600E^ mutation of PTC, and constructed a comprehensive preoperative nomogram for the prediction of the BRAF^V600E^ gene, with the aim to provide information for individual treatment decision-making.

## 2. Materials and Methods

Our retrospective study was conducted in Chongqing General Hospital (Chongqing, China) and was approved by the Institutional Ethics Committee of Chongqing General Hospital (KY S2023-002-01). We followed the ethical guidelines in the Declaration of Helsinki. With the permission from the ethics committee, patients were not required to sign informed consent forms owing to the retrospective study, and their private information was strictly protected in the medical record system.

### 2.1. Patient Selection

**Figure 1:**
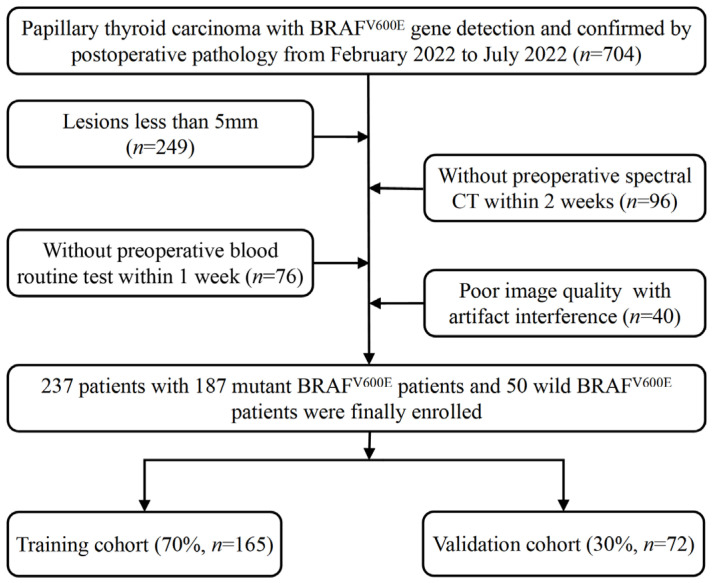
Flow chart of the patient recruitment pathway in this study.

Consecutive patients with pathologically confirmed PTC and BRAF^V600E^ detection were reviewed from February 2022 to July 2022. The inclusion criteria were as follows: (1) PTC confirmed via postoperative pathology; (2) the BRAF^V600E^ mutation detected from the surgery or fine needle aspiration (FNA); and (3) patient had undergone DLCT scan prior to thyroidectomy. The exclusion criteria were as follows: (1) lesions less than 5 mm in diameter; (2) absence of the blood routine examination or liver function test, and (3) poor image quality with artifact interference. Under the criteria, 704 patients were identified, and 467 patients were excluded (as shown in Figure 1). Ultimately, the study population was composed of 237 patients.

### 2.2. DLCT Imaging Acquisition

All patients in this study acquired unenhanced and contrast-enhanced neck images using the IQon spectral CT scanner (a 64-slice DLCT device, Philips Healthcare, Amsterdam, The Netherlands). The contrast-enhanced examinations were carried out using a nonionic contrast media injection (Iodixanol; 320 mgI/mL). The CT scans were carried out ranging from the base of the skull to the aortic arch with the following details: a tube voltage of 120 kV; a tube current controlled by automated exposure control function; a field of view of 350 × 350 mm; a detector collimation of 64 × 0.625 mm; a matrix of 512 × 512; a slice thickness of 0.625 mm; and a reconstruction thickness of 3 mm. The injection rate was 3.5 mL/s with the dose of 1.5 mL/kg, followed by a saline solution of 30 mL at the same rate. A circular region of interest (ROI) was put in the descending aorta lumen for scan triggering at the tracheal bifurcation level. The contrast-enhanced scan was triggered when the CT value in the ROI reached 150 Hounsfield units (HU).

### 2.3. AEF and DLCT Parameters Measurement

The AEF was calculated using the formula $AEFS=\frac{ICa}{ICv}$ where ICa and ICv represent the IC value on the arterial and venous phase images, respectively. The quantitative parameters of DLCT were IC, normalized IC (NIC), Zeff, and the slope of spectral HU curve (λ_HU_) in the arterial phase, and IC in the venous phase (ICv). All the quantitative analysis of contrast-enhanced images was performed using the vendor’s spectral post-processing workstation (IntelliSpace Portal Version 10.1, Philips Healthcare, Amsterdam, The Netherlands). The detailed workflow acquiring the quantitative parameters of dual-energy CT was as follows: First, the iodine-concentration-based reconstruction images, the virtual monoenergetic images, and effective atomic number images were reconstructed for each contrast-enhanced DLCT scan using the raw data. Second, on the cross-sectional images, a circular ROI was manually drawn as large as possible to cover the largest solid portion of the PTC lesion, avoiding necrosis, calcification, and cystic areas. For multiple lesions, the ROI was drawn on the biggest lesion. Another ROI was put on the central part of the common carotid artery at the same slice. The copy-and-paste function kept the shape, size, and location of ROIs constant on the reconstructed multiparametric images. Finally, the Zeff, λHU, iodine concentration (ICa), and NIC in the arterial phase were generated. The CT values (HU) were, respectively, measured on the 40 keV and 80 keV monoenergetic images. The Zeff and ICa were measured on the effective atomic number images and iodine-concentration-based reconstruction images, respectively. The NIC = ICa of the lesion/IC of the common carotid artery. The λHU = (CT value at 40 keV − CT value at 80 keV)/(80 − 40). After the measurement in the arterial phase, ICs in the venous phase (ICv) were generated on the iodine-concentration-based reconstruction images in the venous phase. Every measurement was carried out three times, and the DLCT parameters were determined by means of the three values. All the measurements were obtained by a senior radiologist with 10 years of experience in head and neck radiology. The patients’ clinical information is concealed for the observer.

The CT image features of PTC, including the maximum diameter, quantity, location of lesions (solitary lesion unilaterally, multiple lesions unilaterally, and multiple lesions bilaterally), and calcification (including microcalcification, macrocalcification, and peripheral calcification), were obtained.

### 2.4. Detection of the BRAF^V600E^ Mutation and Collection of Clinical Information

For detection of the BRAF^V600E^ mutation, genomic DNA was derived from primary PTC tissue and the exon 15 of the BRAF gene was sequenced as described previously [20].

Baseline clinical data, including gender, age, nodular goiter, Hashimoto’s thyroiditis (HT), inflammatory markers including neutrophil/lymphocyte ratio (NLR), systemic inflammation response index (SIRI), and prognostic nutritional index (PNI), were collected from medical records.

### 2.5. Prediction Models Based on Significant Parameters and Construction of Nomogram

After the univariate analysis, the significantly different parameters were selected for the multivariate logistic regression (LR) analysis. Prediction models were developed based on available variables in different situations using the training set and then were validated using the validation set. The diagnostic performance of these models was analyzed using the receiver operating characteristic (ROC) curves and the corresponding areas under the curves (AUCs). Furthermore, a nomogram based on the model with the highest AUC in the validation cohort was constructed as a visual tool to improve the applicability of the prediction model in clinical practice.

### 2.6. Statistical Analysis

SPSS (version 25.0, IBM, USA) and R software (version 4.1.0, http://www.R-project.org) were used to perform the statistical analyses. The two independent sample *t* test or the Mann–Whitney U test was selected based on the normality and variance homogeneity of the continuous data. The categorical data were analyzed using chi-square test. The diagnostic performance of the models, based on multivariate LR analysis, was analyzed using the ROC curves. The calibration curve assessed the goodness of fit of the constructed nomogram, while the DCA was used to determine the net benefit of the nomogram. A *p*-value less than 0.05 represents statistical significance.

## 3. Results

### 3.1. Patient Characteristics

Among the included patients, 194 were female (aged 14 to 71 years, with mean age of 42.80 ± 12.62 years) and 43 were male (aged 22 to 59 years, with mean age of 39.52 ± 10.72 years). The rate of HT in this study was 38.8% (92/237). Multiple lesions unilaterally occurred in 11.0% (26/237) of patients and multiple lesions bilaterally occurred in 21.1% (53/237) of patients. The diameter of the included PTC lesions was 10.58 ± 5.31 mm (5–30 mm). The mutation rate of BRAF^V600E^ was 78.9% (187/237).

### 3.2. Comparison of the AEF and DLCT Parameters

The univariate analysis of the clinical information, the AEF and DLCT parameters between the mutant BRAF^V600E^ and wild-type BRAF groups, are summarized in Table 1. The DLCT parameters, including λ_HU_, NIC, and Zeff in the arterial phase, and IC in the venous phase, as well as the AEF, were statistically different between the two groups (*p* < 0.05). The proportions of HT and calcification were higher in the wild group than those in the mutant group (*p* < 0.05).

After the univariate analysis, the significantly different variables (*p* < 0.05) were selected for the multivariate LR analysis. In addition, the IC in the arterial phase (ICa, *p* = 0.082) was also included for the LR analysis.

### 3.3. Construction and Validation of Prediction Models Base on Different Combinations

**Figure 2:**
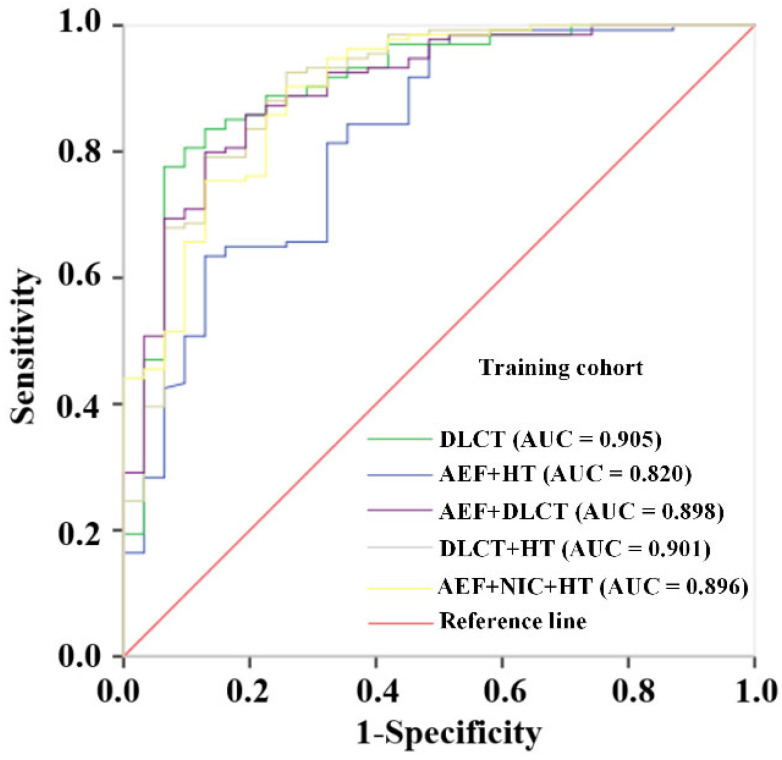
Receiver operating characteristic curves of the prediction models with different combinations of variables for predicting the BRAF^V600E^ mutation in PTC in the training cohort. AEF, arterial enhancement fraction; DLCT, dual-layer detector spectral computed tomography; HT, Hashimoto’s thyroiditis; and NIC, normalized iodine concentration.

**Figure 3:**
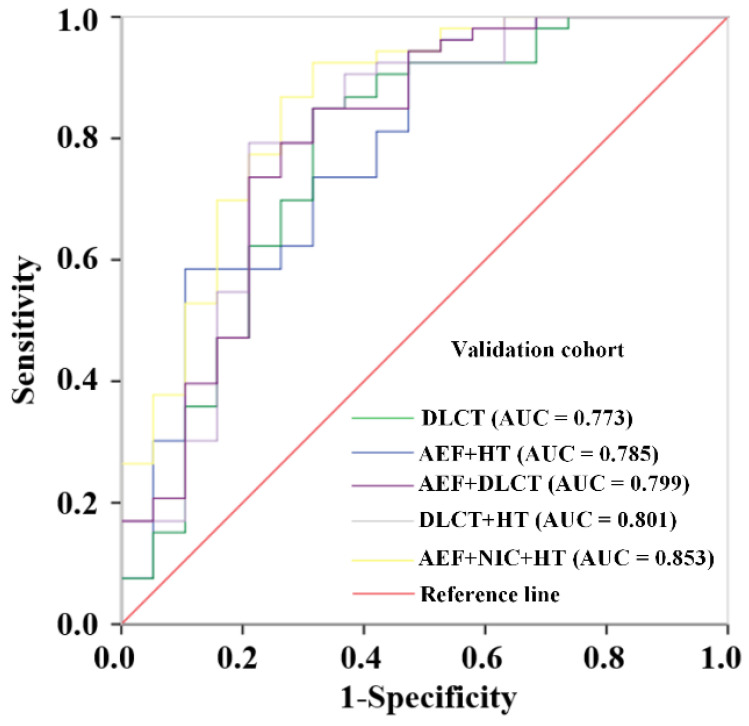
Receiver operating characteristic curves of the prediction models with different combinations of variables for predicting the BRAF^V600E^ mutation in PTC in the validation cohort.

The prediction models based on different combinations of variables were constructed. The diagnostic effectiveness of the models is shown in Table 2. The ROC curves of these models are shown in Figure 2 for the training set and Figure 3 for the validation set. After multivariate LR analysis, the NIC, ICa, ICv, and Zeff were selected to construct the DLCT model. The AEF + DLCT model was constructed using the AEF, NIC, ICv, and Zeff. The DLCT + HT model was constructed using the NIC, ICa, ICv, and HT. However, the comprehensive model finally consisted of AEF, NIC, and HT after all the variables were included in the LR analysis. The formulae of the models are presented in Table S1 in the Supplementary Materials. Among all the models, the comprehensive model presented stable effectiveness and the highest AUC in the validation cohort, with an AUC of 0.896 (95% CI, 0.833–0.959) in the training cohort and 0.853 in the validation cohort. The combination of DLCT and HT also showed a favorable AUC both in the training cohort (0.901, 95% CI, 0.835–0.967) and validation cohort (0.801, 95% CI, 0.669–0.934).

### 3.4. Construction and Evaluation of the Nomogram

**Figure 4:**
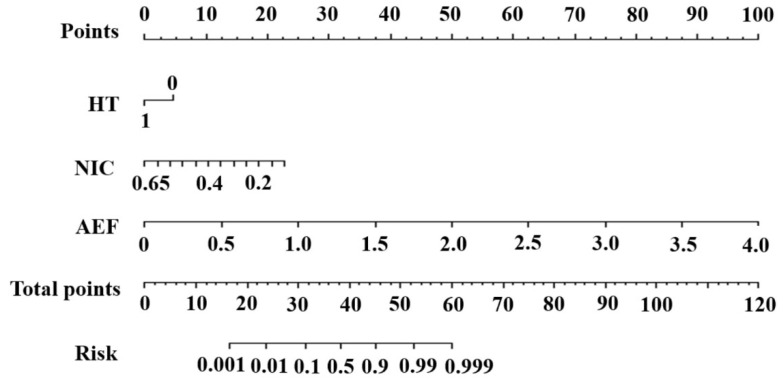
The nomogram was constructed based on the model with the highest area under the receiver operating characteristic curve in the validation cohort.

The nomogram (Figure 4) was constructed based on the comprehensive model. The significantly different parameters were multiplied by their respective regression coefficients, as follows:

$$logit\left(P\right)=-2.182+7.957*AEF-13.148*NIC-1.446*HT$$

**Figure 5:**
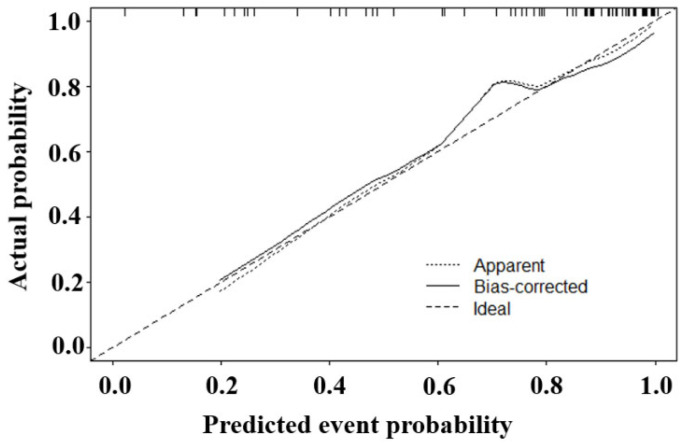
The calibration curve for the constructed nomogram.

**Figure 6:**
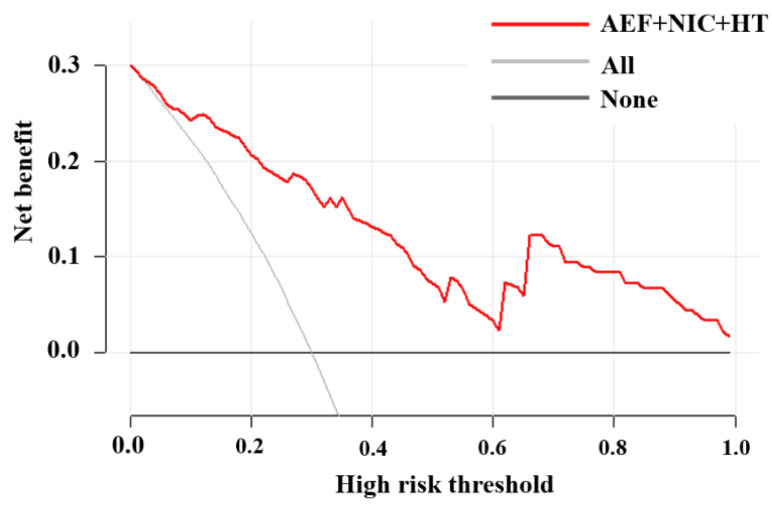
Decision curve analysis for the nomogram. The y-axis measures the net benefit and the x-axis represents the threshold probability.

The prediction results were in good agreement with the actual observations demonstrated by the calibration curve (Figure 5). The DCA showed that this model had a greater net benefit than the all or none intervention strategy, for all the threshold probabilities (Figure 6).

## 4. Discussion

In this study, multiple preoperative prediction models of the BRAF^V600E^ mutation in PTC were established and validated using available variables in different situations. Among the models, the comprehensive model, composed of the AEF, NIC, and HT, exhibited a good and stable performance in the training and validation cohorts. The three variables could be easily measured or acquired both by radiologists and clinicians, which indicated that the model could be an operable tool in clinical reality. As far as we know, this study represents the first development of AEF-DLCT-based model for the prediction of the BRAF^V600E^ mutation in PTC.

The IC derived from dual-energy CT reflects the blood supply and the iodine absorption of the cells in the measured tissues. The AEF derived from dual-energy CT is the ratio of the ICa and ICv which generated from blood supply and iodine absorption of the tissues during the arterial phase and the venous phase. As for blood supply, a previous study has revealed the correlation between the AEF and perfusion index, and that both of them correlated with the histologic findings of tumor vessels [21]. In existent studies, the AEF was mainly used in diagnosing and predicting the treatment response of hepatic diseases based on the increased arterial input of the tumor [10]. Although the BRAF^V600E^ mutation promotes angiogenesis in myopericytoma, and the microvascular density was also higher in BRAF^V600E^-mutant colorectal cancer [12,22], the angiogenesis studies were inconsistent for BRAF^V600E^-mutant thyroid cancer. Husain et al. reported microvascular endothelial cells’ enrichment in BRAF^V600E^-ATC, while Durante et al. demonstrated that proangiogenic molecular decreased in BRAF^V600E^-mutant thyroid cell lines [23]. In this study, the AEF value is higher in the BRAF^V600E^-mutant PTCs than those in the wild type BRAF lesions. Increased arterial input or decreased venous supply, or even both of them to some extent, contribute to this result. More basic studies are needed to explore the microenvironment in PTC with the BRAF^V600E^ mutation.

NIC was used widely in studies for the minimization of the individual circulation-induced influence. The uptake ability of iodine relies on the iodide-metabolizing system of the thyroid cells. The sodium/iodide symporter (NIS) on the membrane of the cells transports iodide into thyroid cells from the blood stream. In the follicle, iodine is oxidized and incorporated into tyrosine residues to form thyroid hormones with the molecules, including thyroglobulin, thyroperoxidase (TPO), and thyroid, stimulating the hormone receptors involved. Previous articles revealed that the BRAF^V600E^ mutation was associated with decreased expression of TPO, NIS, and thyroglobulin in PTCs [24]. The expression of BRAF^V600E^ in thyroid cells led to some iodide-metabolizing genes’ silencing [25]. Thus, the molecular mechanisms of the BRAF mutations mentioned above may be the explanation for the NIC being lower in patients harboring the BRAF^V600E^ mutation.

HT belongs to autoimmune inflammation, with the prevalence being 7.5–11.4% in adults [26]. Mononuclear infiltration and fibrosis gradually replace normal tissues in thyroids with HT. A meta-analysis including 39 original studies revealed that PTC concurrent with HT presented a lower incidence of the BRAF^V600E^ mutation, LN metastasis, and recurrence, which indicated HT as being a potentially protective factor for PTC prognosis [27]. As for the relationship between HT and the BRAF^V600E^ mutation in PTC, some studies found that HT was negatively related to the BRAF^V600E^ mutation, which is in agreement with this study [28,29]. The mechanism of the relationship between HT and the BRAF^V600E^ mutation in PTC remains unclear and further investigation is required to explain that.

However, the current study has several limitations. First, this is a single-center retrospective analysis and the sample size is relatively small. The external multicenter validation cohort is needed to further validate the performance of the models. Second, the pathophysiological or molecular mechanisms correlating the three preoperative variables, the AEF, NIC and HT, with the BRAF^V600E^ mutation in PTC in the prediction model were not explored in this study. While existing studies have suggested potential mechanisms for lower NIC in the BRAF^V600E^ mutant PTC, the mechanisms of the AEF and HT associated with the BRAF^V600E^ mutation in PTC require further pathophysiological or molecular investigations. Third, we ignored the influence of FNA taken before the DLCT examination on the quantitative parameters. The aspiration changed the initial distribution of tissues and may lead to apparent edema and hemorrhage, which would affect the CT value and iodine concentration. Further research exploring the influence of FNA on the quantitative parameters of DLCT is needed. Fourth, this study only included image features which could be easily identified by clinicians and residents like calcification, size, CT value. Agyekum EA et al. found that machine-learning-based US models performed well in identifying the BRAF^V600E^ mutation in PTC, with the best AUC being 0.98 [30]. Better prediction efficiency may be achieved if radiomics or machine learning are combined into the nomogram in this study. Finally, the AEFs in this study were calculated using the ratio of IC in the arterial phase to that of the venous phase, which was adopted by some researchers, rather than the ratio of the attenuation increment, as the AEF firstly introduced. AEFs generated from the two methods were demonstrated as correlating well and exhibiting comparable performance in the diagnosis of cervical lymph node metastasis in PTC [11]. However, the calculation of AEFs in this study reduced the measurement for CT values, while the ICs in the arterial and venous phases are available.

## 5. Conclusions

Among all the prediction models for the BRAF^V600E^ mutation in PTC based on significantly different variables, the DLCT + HT and AEF + DLCT + HT models showed favorable results, both in the training cohort and validation cohort. The comprehensive model finally consisted of the AEF, NIC, and HT, after all the variables were included using LR analysis and presented good and stable effectiveness. In conclusion, we proposed an AEF-DLCT-based preoperative prediction model with three noninvasively obtained variables, the AEF, NIC, and HT, in this study, which could assist clinicians in making personalized treatments.

## Figures and Tables

**Table 1:** Univariate analysis between the mutant and wild BRAF^V600E^ gene cohorts.

Variables	Wild Set (*n* = 50)	Mutant Set (*n* = 187)	*p*-Value
Gender			0.976
male	9	34	
female	41	153	
Age			0.491
<40	26	87	
≥40	24	100	
Laterality			0.124
solitary lesions unilaterally	39	122	
multiple lesions unilaterally	6	20	
multiple lesions bilaterally	5	45	
HT			0.002
negative	21	124	
positive	29	63	
Calcification			0.034
negative	28	134	
positive	22	53	
Diamater (mm)	8.00 (6.00, 12.25)	9.00 (7.00, 13.00)	0.39
AEF	0.90 (0.70, 1.09)	1.17 (1.02, 1.39)	<0.001
DLCT parameters			
λHU	5.27 ± 3.12	4.12 ± 1.25	0.013
NIC	0.30 (0.24, 0.48)	0.27 (0.21, 0.34)	0.010
Zeff	8.79 ± 0.61	8.54 ± 0.36	0.006
IC_a_	2.78 ± 1.21	3.11 ± 0.96	0.082
IC_v_	3.02 ± 0.97	2.54 ± 0.75	<0.001
Tumor-associated inflammatory parameters			
NLR	2.29 (1.75, 3.01)	2.06 (1.67, 2.81)	0.395
SIRI	0.67 (0.52, 0.92)	0.64 (0.43, 0.83)	0.760
PNI	51.50 ± 3.88	51.33 ± 4.04	0.789

AEF, arterial enhancement fraction; DLCT, dual-layer detector spectral CT; HT, Hashimoto’s thyroiditis; NLR, neutrophil/lymphocyte ratio; PNI, prognostic nutritional index; and SIRI, systemic inflammation response index.

**Table 2:** Diagnostic effectiveness of the constructed models in the training and validation cohort.

Models	Training Cohort			Validation Cohort		
	AUC (95% CI)	Sensitivity	Specificity	AUC (95% CI)	Sensitivity	Specificity
DLCT	0.905 (0.841–0.968)	0.806	0.903	0.773 (0.633–0.912)	0.849	0.684
AEF + HT	0.820 (0.733–0.906)	0.634	0.871	0.785 (0.656–0.913)	0.943	0.526
AEF + DLCT	0.898 (0.837–0.959)	0.799	0.871	0.799 (0.669–0.929)	0.849	0.684
DLCT + HT	0.901 (0.835–0.967)	0.925	0.742	0.801 (0.669–0.934)	0.792	0.789
AEF + NIC + HT	0.896 (0.833–0.959)	0.903	0.742	0.853 (0.746–0.961)	0.925	0.684

AEF, arterial enhancement fraction; AUC, area under the curve; DLCT, dual-layer detector spectral CT; HT, Hashimoto’s thyroiditis; and NIC, normalized iodine concentration.

## Data Availability

The data presented in this study are available on request from the corresponding author. The data are not publicly available due to privacy restrictions.

## Supplementary Materials

The following supporting information can be downloaded at: https://www.mdpi.com/article/10.3390/diagnostics15212817/s1. Table S1: The prediction models based on available variables in different situations.
