# Supplementary material for: Arterial Enhancement Fraction-Spectral CT-Based Model as Part of Prediction Model in BRAFV600E-Positive Papillary Thyroid Carcinoma
Source: Diagnostics (Basel). 2025 Nov 6;15(21):2817. doi: 10.3390/diagnostics15212817 (PMC12607739; doi:10.3390/diagnostics15212817)
Supplement: Supplementary file 1 [file diagnostics-15-02817-s001.zip › diagnostics-3942361-supplementary.pdf]

Prediction of BRAF^{V600E} mutation in papillary thyroid carcinoma by an arterial enhancement fraction-spectral CT based model

Results

Construction and validation of prediction models base on different combinations

Table S1 The prediction models base on available variables in different situations.

Models	Formulas
DLCT	logit P =17.901-10.838*NIC+3.374*ICa-2.432*ICv+1.871*Z
AEF+HT	logit P =-3.926+5.476*AEF-0.982*HT
AEF+DLCT	logit P =-13.390*NIC+9.357*AEF-0.742*Z+0.784*ICv
DLCT+HT	logit P =4.945-15.449*NIC-3.169*ICa-2.620*ICv-1.402*HT
AEF+NIC+HT	logit P =-2.182+7.957*AEF-13.148*NIC-1.446*HT

NIC, normalized iodine concentration; ICa, IC in the arterial phase; ICv, IC in the venous phase; Zeff, effective atomic number; HT, Hashimoto's thyroiditis; AEF, arterial enhancement fraction.
